# A robust hydroponic system for horticulture farming using deep learning, IoT, and mobile application

**DOI:** 10.1371/journal.pone.0330488

**Published:** 2025-09-08

**Authors:** Nadim Nawshad, Md. Asraf Ali, Ku Nurul Fazira, R. Badlishah Ahmad, Mejbah Ahammad, Nasim Ahmed

**Affiliations:** 1 Department of Computer Science, American International University - Bangladesh, Dhaka, Bangladesh; 2 Artificial Intelligence Research and Innovation Lab - AIRIL, Dhaka, Bangladesh; 3 Faculty of Electronics Engineering and Technology, Universiti Malaysia Perlis (UniMAP), Perlis, Malaysia; 4 School of Computer Science, The University of Sydney, Sydney, New South Wales, Australia; Adani University, INDIA

## Abstract

Due to limited literacy among root-level farmers, hydroponic farming in Bangladesh faces significant challenges. Therefore, there is a demand for easy-to-use technical systems to help farmers to monitor and operate smart systems. To address the issue, this study introduces a robust hydroponic system that provides automatic guidelines, monitoring, and a disease detection system. The main objective of this paper is to support farmers by making the cultivation process more convenient and less stressful. The system is structured into three phases: hardware implementation using WeMos controllers, disease detection using the Deep Learning model, and mobile application development for sensor data analysis and automatic notifications. The proposed system significantly demonstrates a high disease detection accuracy of 98.5%. Moreover, the survey report shows that around 80% of the root-level farmers find the system helpful for their cultivation process and increase the usability and monitoring of the system. These findings suggest that the proposed system can substantially improve the operational efficiency and sustainability of hydroponic farming, and it has the potential to enable more effective resource management and disease prevention strategies.

## Introduction

As the world population continues to grow, food production demand has also increased. According to a press release of the United Nations, the world population is projected to reach 8.6 billion by 2030, which creates a shortage of cultivable land [1]. Climate change is also a factor that has an impact on agricultural land [2]. A study by World Health Organization estimated that approximately 2.3 billion people (around 29.3% of the global population) experienced moderate to severe levels of food insecurity [3]. To reduce food insecurity, hydroponics in horticulture have a beneficial effect on food supply by promoting the growth of green spaces [4,5].

Numerous researchers have [6–8] applied horticulture farming to enhance the cultivation of plants to produce food, medicine, or aesthetic appeal. Hydroponic farming is an innovative farming approach to growing plants without soil. It has the potential to revolutionize the agriculture sector, particularly in urban and arid areas. At present, different types of hydroponic cultivation methods exist, including the Nutrient Film Technique (NFT), Deep-Water Culture (DWC), Ebb Flow, wick, and drip systems. In horticulture gardening, NFT and DWC are commonly used [9].

In Bangladesh, farming still remains the primary source of income for majority of the population [10]. However, the adaptation of hydroponic farming faces challenges due to the limited literacy among the root-level farmers and lack of knowledge [11]. The majority of the farmers need help with the complexity of monitoring and operating the smart hydroponic system. These technological gaps demotivate them from implementing and adapting smart hydroponic systems.

Existing smart hydroponic systems are primarily designed for large-scale farming. These systems are typically used by farmers who are literate and have knowledge of advanced technologies. In fact, the adoption of such systems often necessitates extensive training to operate and monitor effectively [12,13], which is not feasible for the majority of Bangladeshi farmers. Real-time monitoring with immediate feedback is crucial for low-literacy users to maintain the smart system effectively. However, existing systems frequently ignore this feature [14]. Leaving farmers without necessary guidance make them helpless to address issues promptly. Moreover, a user-friendly application with an advanced disease detection and solution-providing system plays a vital role in the accurate and timely detection of plant disease and taking further steps against the disease. Many current systems do not integrate advanced disease detection technologies with solution-providing features that are dedicated to horticulture farming. Additionally, existing systems do not develop mobile applications that consider the root-level farmer’s needs, which makes a system unknown and difficult to navigate. This results in operational challenges and user frustration.

Considering these challenges, there is a need for a smart hydroponic system that can resolve the specific requirements of Bangladeshi farmers, including user-friendly application, real-time monitoring, guideline feature, disease detection and solution-providing feature. By addressing these issues, the adoption and operation of the smart hydroponic system would be more comfortable. Therefore, this study introduces an IoT-based, robust, user-friendly hydroponic system for horticulture farmers, including an exclusive guideline-provider system and a specialized disease detection system for horticulture users. It is divided into three phases. The first phase is the implementation of an IoT environment, including necessary sensors in a hydroponic environment. The second phase is the disease detection phase, where a convolutional neural network (CNN), HCNet, is trained and tested on a comprehensive dataset collected from controlled hydroponic environments to detect the disease and provide probable solutions. The third phase is the implementation of an easy-to-use mobile application to monitor the system and provide effective guidelines for better cultivation. The main contributions of this paper are follows:

- Development of IoT environment to grow vegetables in horticulture using DWC technique.
- Development of an easy-to-use mobile application for providing guidelines by analyzing sensor data and presenting sensor data to the system.
- Development of an advanced DL model, HCNet, for detecting disease and providing probable solutions for that disease.

The rest of the paper is organized as follows. Section Related works, which provides a description of related works. Section Materials and methods provides structures and comprehensive descriptions of each category. Section Results and discussion provides results and discusses each environment’s activities. Moreover, Conclusion will summarise the work and discuss future work.

## Related works

Soilless cultivation, such as like hydroponic [15–17] and aeroponic [18–20], are viable methods to cultivate nutritious vegetables. With technological advancements, soilless cultivation requires fewer resources, which has replaced traditional cultivation. This transition results in a continuous cycle of production, enhanced yield considering climate and weather limitations, automation, labour reduction, more plans per unit area, lower water consumption, and higher seeding survival rate [21–25].

### IoT environment monitoring and guideline providing.

The implementation of IoT requires much-advanced technologies [26]. Recent advances in several sectors of IoT are heavily influenced by monitoring systems [27–31]. Mobile application based monitoring [32–36] is becoming increasingly popular. A user can monitor the environment by wireless communication and get notified about any anomaly. To notify about any anomaly in the system, most of the researchers used SMS notification [37,38], LED display [39–41], bot [42] and application based notification [43–45]. However, state-of-the-art performance on hydroponic monitoring mobile applications lack features that could simplify the cultivation process, including providing guidelines such as suitable day and night temperature, pH level, and water level, plant nursing descriptions, a history of all the features, and symbolic indications with written instructions for illiterate farmers.

### Disease detection and solution providing.

The majority of Bangladesh’s farmers are not sufficiently equipped with advanced agricultural technologies to detect plant disease [46]. As a result, they usually depend on their visual inspection, which is time-consuming and not always accurate [47]. To overcome this issue, several researchers utilized Machine Learning (ML) and DL techniques [48–50]. To train a model, many of the researchers used the PlantVillage dataset [51–55] because of its large size. Although, the entire dataset is unsuitable for horticulture in a hydroponic environment. In this work, we merged the PlantDoc and PlantVillage datasets and used only suitable plants for the training. Additionally, we provide potential solutions, which is missing in the existing works, so that the farmer can take further action against the disease.

Based on the literature reviews above, we note that the existing researchers have limitations in providing nursing guidelines, an easy-to-use application for root-level farmers and an advanced disease detection system using a large dataset specifically targeted for horticulture farming with a solution-providing feature. In this context of hydroponic in horticulture, we proposed a user-friendly hydroponic system that is easy to use for root-level farmers, provides guidelines to the user, and incorporate an advanced disease detection model (HCNet) using a merged dataset with a solution-providing feature. Table 1 provides an overview of state-of-art systems.

**Table 1:** Research on smart horticulture.

Reference	IoT	hydroponic implementation	disease detection	disease solution provide	mobile application	guideline provide	cost
[56]	Yes	No	No	No	No	No	-
[57]	Yes	No	No	No	No	No	64.5
[58]	Yes	Yes	No	No	Yes	No	68
[59]	Yes	Yes	No	No	No	No	65
[60]	Yes	Yes	Yes	No	No	No	27.5
[61]	Yes	Yes	Yes	No	No	No	150
[62]	Yes	Yes	Yes	No	Yes	No	72
[63]	Yes	Yes	Yes	No	Yes	No	720

## Materials and methods

### Hydroponic setup

**Fig 1:**
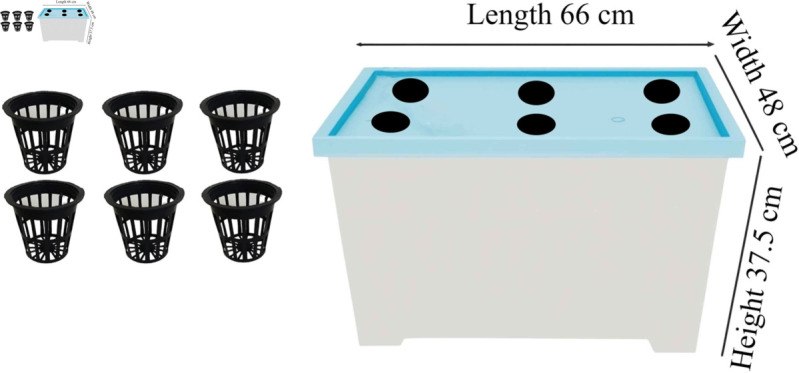
DWC setup of the system.

For experimental purposes, we used six tomato plants. We utilized six cylindrical pots to hold coco peat. Coco peat not only helps the plant absorb the nutrients but also holds up the tree. We nested the pots in the holes of a nutrient reservoir box so that the coco peat could absorb the nutrition solution. The length of the nutrient reservoir box is 66cm, the width is 48 cm, and the height is 37.5cm (Fig 1). We used a mixture of Nitrogen, Phosphorus, and Potassium as the nutrient solution in the system.

### Architecture of the IoT environment

**Fig 2:**
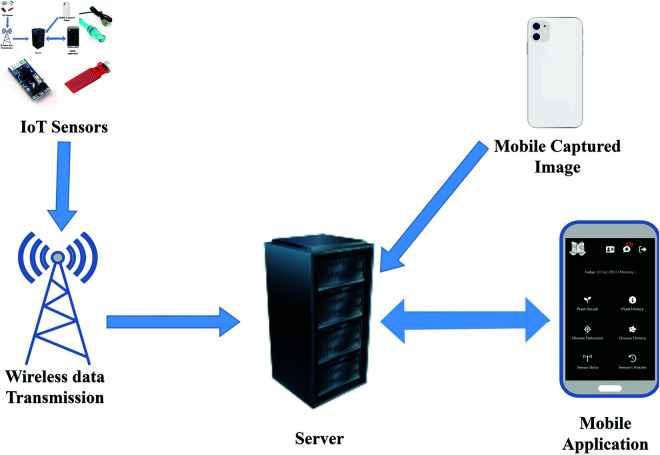
Block diagram of the system.

We designed the proposed system by integrating IoT with mobile application (Fig 2). The figure illustrates that the IoT sensors transmit data to the MySQL server. The MySQL server stores sensor data and presents it to the user through mobile application. The system analyses the sensor data to monitor the current condition of the hydroponic environment. The user can use their mobile phone to capture images of affected leaves for disease prediction. The server stores these images to retrain the disease detection model. After predicting the disease, the server sends the disease name and probable solution to the user’s mobile application. Additionally, the mobile application shows the real-time situation of the IoT environment and provides comprehensive details about how to take care of plants. All the sensor data histories, provided guidelines and disease histories are stored on a server for further analysis.

We divided the system into two environments. The first section is the hardware environment, where the sensors and other components are structured in horticulture farming. Another environment is the software environment for managing and monitoring, providing guidelines, and disease detection with solution-provider features.

### Hardware environment

**Fig 3:**
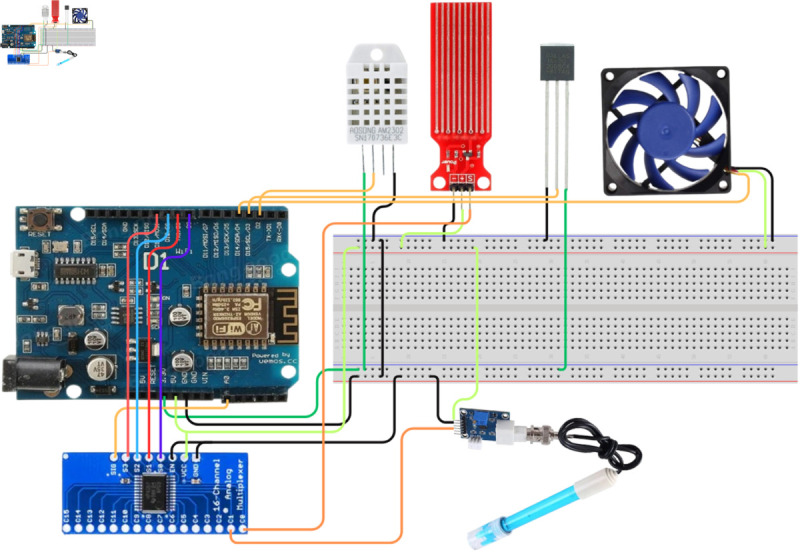
Hardware architecture of the system.

For the hardware environment (Fig 3), we used Aurdino WeMos as the microcontroller. The esp8266 wifi module is integrated with WeMos to establish communication with the application. We used various sensors to collect data to monitor the system and analyze different parameters of horticulture farming. The model extracts the key components, including the temperature of the outside environment and water sensors, water level sensors, and pH sensors, which are necessary for monitoring horticulture farming.

Considering the financial constraints of the root-level farmers, we minimized the cost of the camera module. The system reduces the camera module from the system and alters the feature by using the user’s smartphone. The maximum and minimum limits of temperature adaptability are different for each plant. Therefore, the DHT22 measures the temperature and humidity of an outside environment because the temperature and humidity affect the plant’s healthy growth. DS18B20 measures the temperature of the water. SEN0161 monitors the pH level of water. It extracts information about the nature of the water, whether it is acidic or basic. We used a water level sensor to measure the water level of the system. For water level measurement, we labelled it as 1, 2, 3, 4 and 5, where 1 refers to the minimum, and 5 refers to the maximum. The cooler follows the instructions of the microcontroller to activate or deactivate the fan. Table 2 presents the responsibilities of the sensors.

**Table 2:** List of entities.

Entity	Responsibility
pH sensor	pH sensor is responsible for measuring the pH level of the system. The pH level is measured between 4 and 10, with 0.01 pH resolution and 0.1 pH accuracy.
Temperature and Humidity sensor	It is responsible for measuring the temperature and humidity of the outside environment.
Water temperature sensor	It is responsible for measuring the temperature of the water.
Water level sensor	It is responsible for measuring the water level of the pot. As we are using the DWC technique, it is important to monitor the water level of the pot.
The Arduino WeMos D1 controller	It is responsible for collecting all the sensor data and passing it to the server via wireless communication.

We used Arduino IDE as the compiler to compile the WeMos controller and programmed using C++ to set the logic for sending the sensor data through wireless transmission. The server stores the data with a specific username given by a particular user. The algorithm detailing the communication of the controller is as follows:



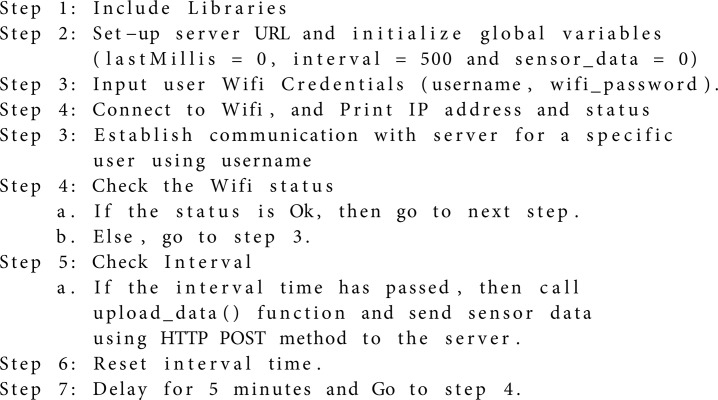



### Database

We used the MySQL database to store individual farming records, various plant nursing information, sensor data, and previous cultivation history. The database also includes information about a plant, such as suitable temperature and humidity (maximum and minimum) of a plant during the day and night, suitable ratio of nutrition for a particular plant, probable solution of each disease, suitable water temperature, and level description.

The database has 16 tables to store information about user information, sensor data, cultivation and disease history, plant information, suitable environment information for each plant, and probable solutions for each disease.

The user information includes the user’s personal information, such as full name, location, and cultivation experience. The sensor data includes temperature and humidity, water temperature, water level, and pH. The information about the environment includes the maximum and minimum temperature, humidity, and pH of both day and night for each plant. We used ten plant’s suitable environment information (Blueberry, Cherry, Grape, Peach, Pepper, Raspberry, Soybean, Squash, Strawberry and Tomato) for this current study. The information on these plants is also stored in a separate table. A table contains the solutions to the diseases that are used to train the model.

### Software environment

**Fig 4:**
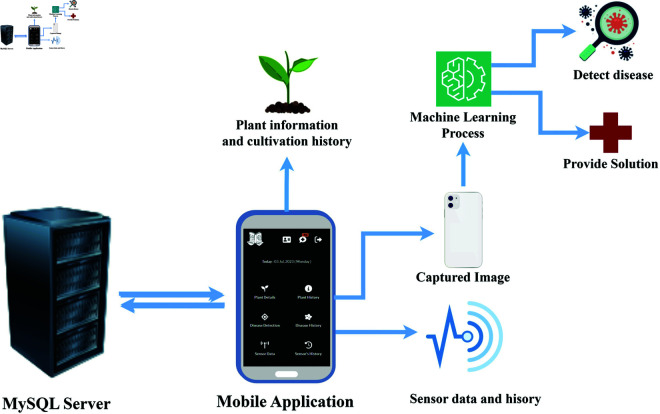
Software architecture of the system.

The software environment (Fig 4) includes an easy-to-use mobile application, DL approaches for disease detection, and a feature that provides guidelines.

#### User-friendly interface.

We built a mobile application to provide a user-friendly interface to the user with all the features, including monitoring and maintaining, disease detection feature and guideline providing feature.

**Fig 5:**
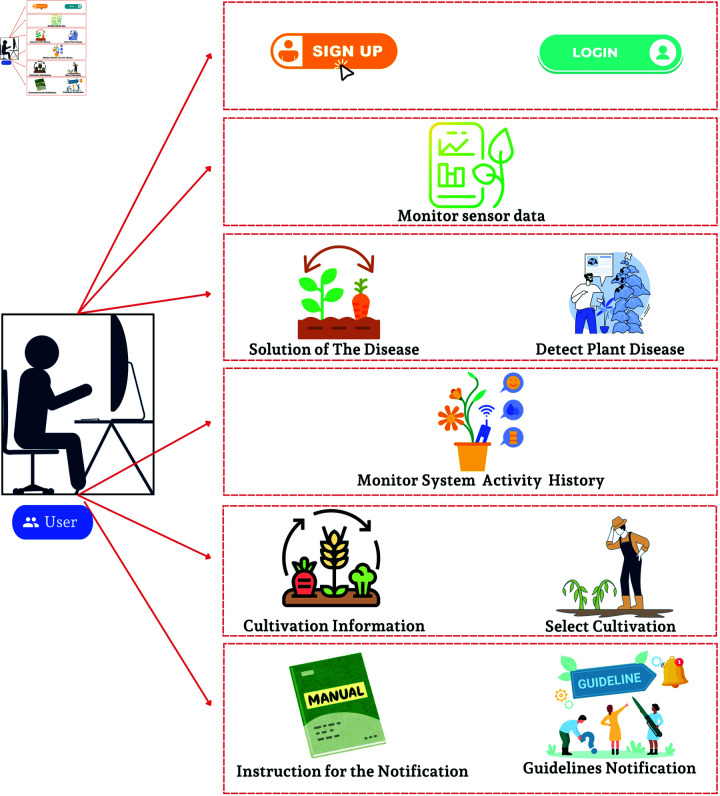
Usecase diagram of the mobile application.

It also gives information about each plant description, cultivation process, and list of disease detection and plant cultivation histories. The use-case diagram (Fig 5) of the mobile application shows that a user has to create an account if he is a new user by setting a unique username and password. For security purposes, we used SHA-256 hashing method to encrypted the password. Then, the user has to login to the system using the username and password to access the dashboard. The dashboard contains six different features, including Plant details, Cultivation History, Live Sensor Data, Sensor Activity, Disease Detection and Disease history.

When a logged-in user select any option of the application, such as disease detection, cultivation history, or live sensor data, the query will extract data for the specific user using his unique username.

To testify the user-friendliness of the proposed system, we surveyed 100 root-level farmers asking their opinion about the application. We showed them the application and asked them to give a rating based on some questions. The questions are:

- How easy was it to navigate through the application?
- Were the icons and buttons intuitive and easy to understand?
- How useful did you find the information provided by the application for your farming activities?
- Did the application help you make better decisions regarding your farming practices?
- Overall, how satisfied are you with the application?

We used a five-point Likert chart to capture the responses. The scale ranged from Very Satisfied to Very Dissatisfied to make the analysis process more convenient.

#### Guideline providing feature.

The provision of guidelines is a significant addition to this study. The live monitoring feature continuously displays the current condition of the hydroponic environment through the mobile application, which is further analyzed to provide guidelines. The sensor data is compared with the specific plant’s ideal environmental data at the back end. If the data crosses the limit of the suitable environment for more than 5 minutes, it will notify the user to take further steps. The notification repeats every 5 minutes until the user responds. The algorithm of notification is given below:



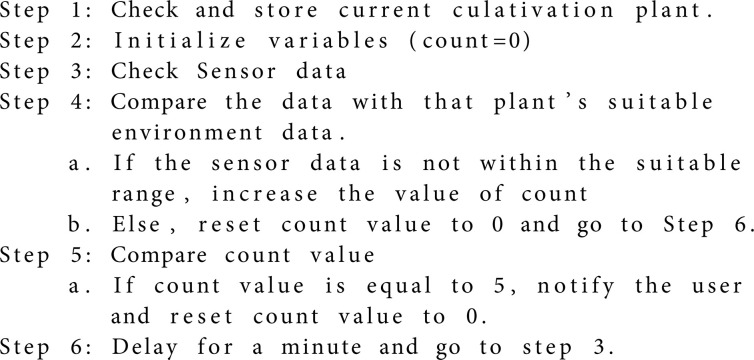



#### Disease detection feature.

Disease detection is the unique feature of mobile application with the help of advance DL model, in this proposed work a new DL model is proposed named HCNet for the disease detection of plant. An activation function softmax is used. A python language is used in order to train the model. It detects the disease as well as it gives the probable solution of that detected disease.

**Fig 6:**
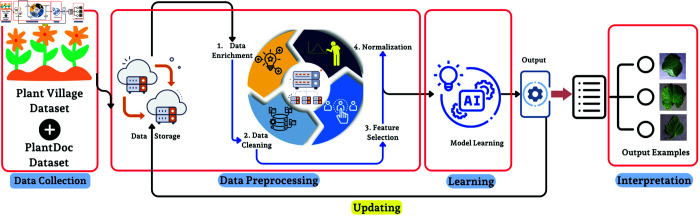
A DL workflow for plant disease identification.

The process of the disease detection feature of the proposed model is detailed in the DL pipeline (Fig 6). The data was collected from Plant Village and PlantDoc. Different stages of data preprocessing were performed i.e. storage, enrichment, cleaning, feature Selection in model training etc. Finally, the results have been shown visually that how model diagnoses the plant health.

#### Dataset

**Fig 7:**
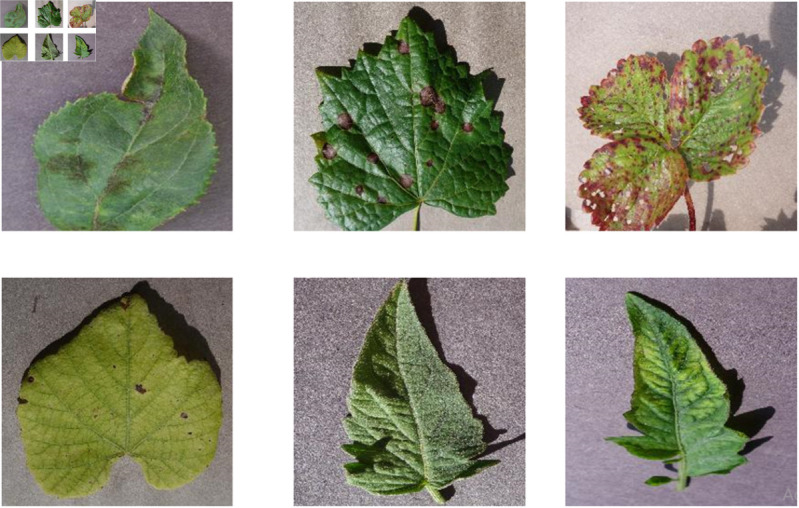
Dataset sample for disease detection.

The dataset includes 27 different classes and more than 42 thousands images (Fig 7). We have merged The Plant Village dataset with the Plant Doc dataset in order to create a new big dataset for training our model. Data is splitted into training, testing and validation set by ratio of 80:10:10.

#### DL process.

**Fig 8:**
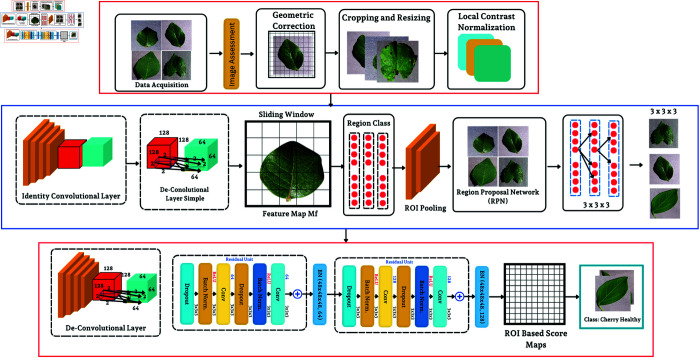
DL pipeline for plant disease detection, from initial leaf image acquisition to the final classification of the leaf’s health status.

This diagram gives the idea of the steps related to stages in a DL pipeline for plant disease detection, ranging from data acquisition to the classification phase (Fig 8). The proposed work starts with the collection of leaf images and then proceeds to preprocessing, i.e., geometric correction to rotate and scale images captured are performed.

Then, images are cropped and resized to a given dimension, followed by local contrast normalization to enhance patterns. Further, a sliding window is convoluted over the feature map for detecting regions of interest, which will be applied by the Region Proposal Network (RPN) via disease-specific proposal generation. Convolution with the deep convolutional network processes each proposal region, generating a score map of regions-of-interest (ROI) that estimates the probability of a proposed leaf region is healthy or unhealthy based on learned characteristics in order to enhance precision in disease identification using image processing and machine learning techniques. The mathematical terms are follows:

- **Geometric correction:** Post-acquisition, geometric correction of images is done to remove any distortion so that the analysis is carried out with an accurate representation of data. It involves an affine transformation and is mathematically represented as:
  $$LetT(x,y)=[\begin{array}{cc} a & b\\ c & d \end{array}][\begin{array}{c} x\\ y \end{array}]+[\begin{array}{c} e\\ f \end{array}]$$ (1)
  where those were *x* and *y* , the original pixel coordinates, where a,b,c,d,e, and *f* are the ones that will normalize the image dimensions and orientations.
- **Local contrast normalization:** Local contrast normalization is used to improve the visibility of pathological features in the images:
  $$I_{x,y}^{\prime }=\frac{I_{x,y}-\mu_{L}(x,y)}{\sigma_{L}(x,y)+\epsilon }$$ (2)
  This step modifies the pixel values with respect to their local mean ($\mu_{L}$) and standard deviation ($\sigma_{L}$). This enhancement step ensures that the lesions, discolouration and any other signs of disease are emphasized for further analysis.
- **Feature extraction using sliding window:** Here, we have been introduced to a method that is related to the corner point detection task, where key points will be detected by this method.
  $$F_{i,j}=\sum_{u=-k}^{k}\sum_{v=-k}^{k}W_{u,v}\cdot I(i+u,j+v)$$ (3)
  This takes advantage of important textural and color changes associated with plant disease symptoms, such as mosaics caused by viral infections or the rust-colored pustules that appear during many fungal infections.
- **Region proposal network (RPN):** The RPN uses these features to determine object proposal regions of the image with a softmax function.
  $$P_{obj}=\sigma \left(\sum_{i}w_{i}\cdot f_{i}+b\right)$$ (4)
  This model can well discriminate between healthy and diseased tissue, and it allows us to make a focused study of the troublesome parts.
- **ROI pooling:** The proposals are then ROIs (Regions of Interest) that apply ROI pooling to their areas in order to have feature inputs with the same size as the classification model.
  $$v_{c,p}=\max_{(x,y)\in \text{region }p}F_{c}(x,y)$$ (5)
  This pooling mechanism makes sure that classifier element inputs are of the same size: the data structure is preserved until the classification step.

After extracting the features, we train a deep convolutional neural network (CNN) to make predictions about the health of those leaves. This network is made up of layers of convolutions, activations, and pooling that learn to recognize patterns in the data for the final task in classifying disease. The output of the system classifies each leaf image as healthy or infected by fungi, bacteria, or viruses. This classification is done using a learned disease signature by model. Finally, the diagnostic is embedded in agricultural management systems and provides crop treatment decision information to farmers to reduce yield losses and improve food security.

**Fig 9:**
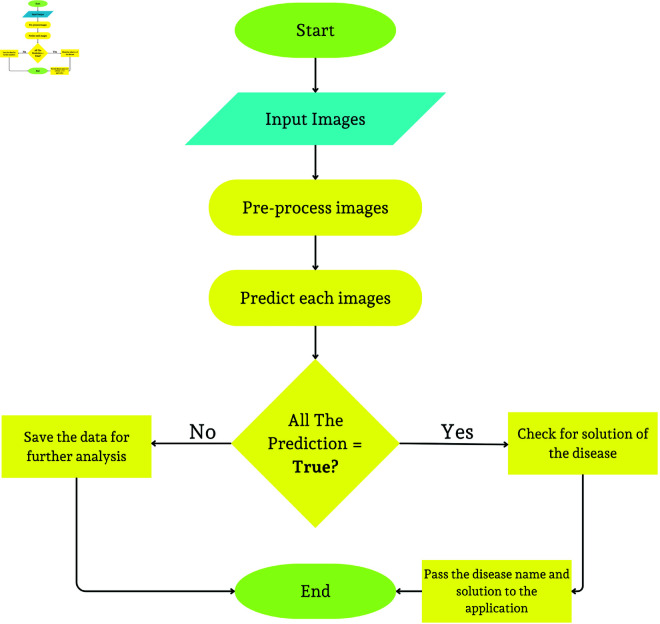
Flowchart for image-based disease prediction process.

The user has to capture four images of the infected leaves through a mobile camera, or he/she can upload pre-captured images for the prediction system. Then, the system will preprocess these uploaded images and compress them according to size. Each image is tested uniquely, and the results are compared if all detections are identical. If the same disease is detected in all four tests of DL, then the token is sent by the DL system on the mobile application along with the name of the disease and suggested solution. The system has a pre-stored suggested solution in its database for each disease, which is based upon the training dataset. The flowchart of predicting diseases describes each process of the steps (Fig 9).

## Results and discussion

The proposed system has shown remarkable results in disease detection and user-friendliness. The WeMos controller successfully communicates with the database by transmitting sensor data. Moreover, the mobile application successfully monitors the system and provides guideline notifications. The result of the proposed HCNet for disease detection feature outperforms other DL models and gives the best accuracy of 98.5%. The solution-providing system also works accurately to provide a solution against the disease.

### Hardware activities

**Fig 10:**
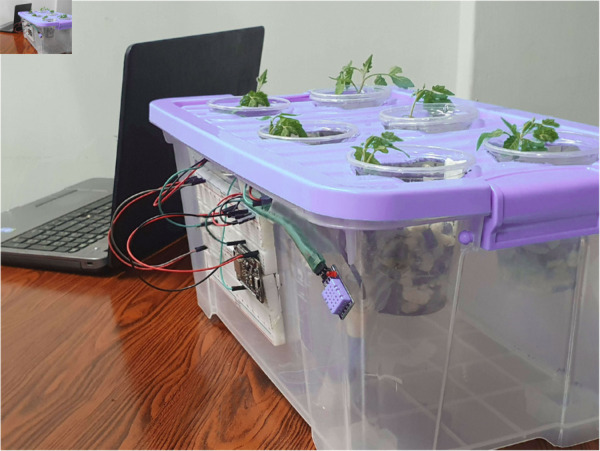
Prototype of the system.

Hardware activities refer to the activities of the IoT system (Fig 10). The WeMos controller successfully collects the data from the sensor and sends it to the server. The pH sensor measures the reading of the liquid’s concentration and keeps sending updates to the server continuously. Additionally, the water level sensor takes care of the water level and notifies the user whenever the water level is high or low. Both the environmental and water temperature sensors successfully measure the temperature. The controller controls the cooler according to the temperature changes.

#### Cost estimation.

The total estimated cost of the system is $57, which is not a burden for the users. Table 3 describes the total cost of the system. It shows that for hydroponic setup, the cost of pots and boxes is $6.5. The sensor cost is $41 in total. For both The controller and cooler cost is $2, and the multiplexer cost is $4.5.

**Table 3:** Cost estimation of current study.

Components Name	Quantity	Cost ($)
SEN0161	1	36
DHT22	1	3
DS18B20	1	1
Aurdino WeMos D1	1	2
Water Level Sensor	1	1
Cooler	1	2.0
CD74HC4067	1	4.5
Cylindrical Pots	6	1.5
Nutrient Reservoir Box	1	5
Total cost		57

Data source: alibaba.com and roboticsbd.com.

### Database activities

**Fig 11:**
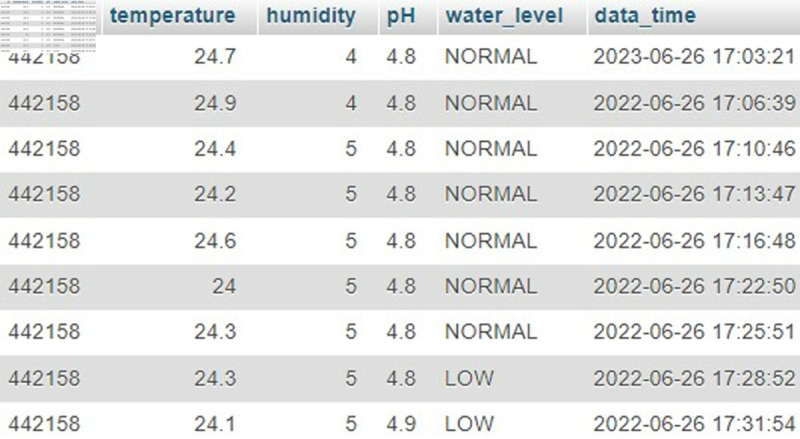
Sensor data stored in a database.

The database is one of the major parts of the system. All the sensor data is successfully stored in the database (Fig 11), and used to provide information to the mobile application. The data is continuously saved to the database after every five minutes.

The input pictures and the description of the detected diseases are stored successfully for monthly dataset retraining. Unpredictable diseases are also stored in the database for further analysis. Moreover, the system retains cultivation history and recent sensor data for analysis, providing supportive notifications to the user. The details of individual plants are also stored in the database and passed to the mobile application successfully to help the beginner avoid initial problems.

### Software activities

#### Mobile application.

We have developed a user-friendly mobile application to meet the needs of farmers. The mobile application successfully visualizes all the features from the MySQL database. The integration of the disease detection performed smoothly. Some key features of mobile applications are as follows:

#### Plant details.

**Fig 12:**
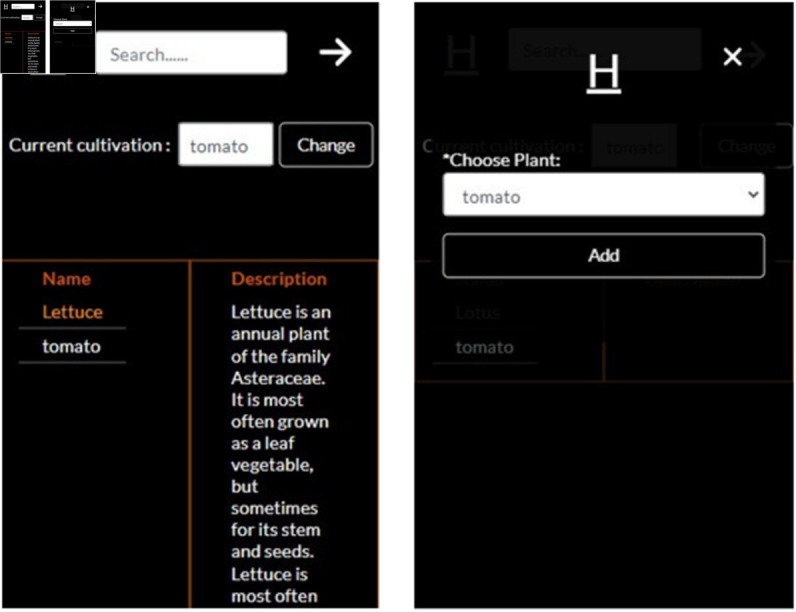
Plant details features.

The Plant Details feature (Fig 12) is mainly responsible to provide details about the plant and give the option to change the plant cultivation options in to the user. The first part serves as a helpful guide for managing different plants, detailing their ideal growing conditions and nutritional needs. The second part helps users set up plant cultivation and offers guidance when they want to start growing new types of plants. It provides flexibility, allowing users to change their cultivation plans as needed.

#### Live sensor monitoring.

**Fig 13:**
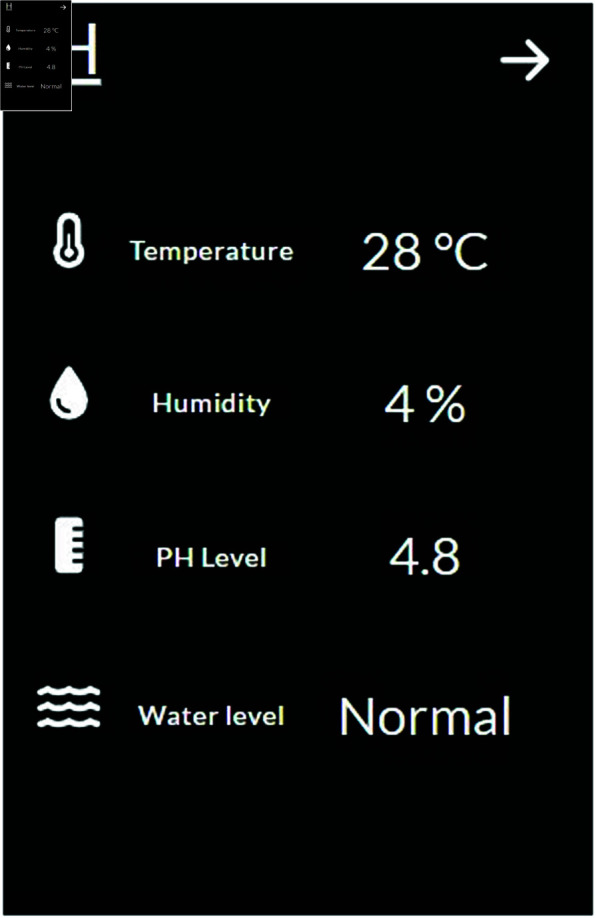
Live sensor data.

The “Live Sensor” option is used to monitor sensor data in real time (Fig 13). The readings come from temperature, humidity, pH, and water level sensors. The cooler controls the temperature of the environment. Water temperature is shown in Celsius, and humidity is shown in percentage. PH sensor reads the pH level, and the Water level is displayed as “High,” “Normal,” or “Low.” The most recent sensor data is refreshed every 5 minutes.

#### History of sensor, cultivation and disease detection.

Cultivation history shows the records of previously cultivated plants. It provides a list of recently cultivated fruits or vegetables and the duration of cultivation. This feature helps a user learn from his previous experiences. Additionally, the application provides sensor data, which helps the user understand how sensor changes affect the plant’s growth. Furthermore, the disease detection history helps a user monitor the plant more conveniently.

#### Guideline providing feature.

**Fig 14:**
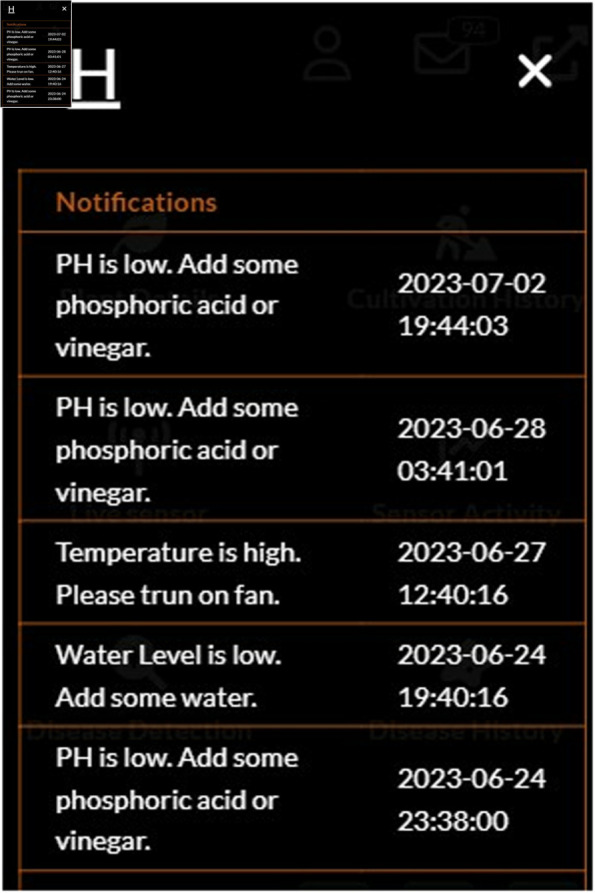
Notification for unwanted situation.

The system guideline option is named as “Notifications” (Fig 14). Through the notifications, the user gets notifications if the sensor’s data are not suitable for the plants. Users can be alert and take further action to maintain a suitable environment by receiving notifications. The notifications are successfully turned off after taking action to maintain a suitable environment.

#### Disease detection and solution providing feature.

**Fig 15:**
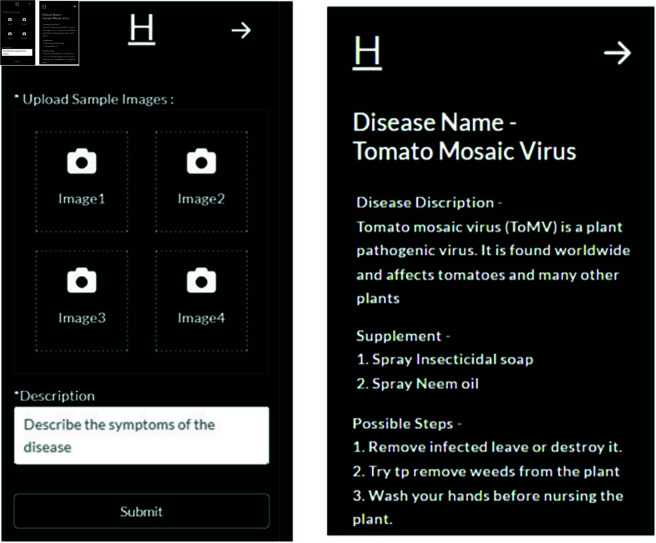
Disease detection and solution providing.

The disease detection feature demonstrates how the system detects the disease of plants and provides probable solutions (Fig 15). Users need to upload four pictures of an affected leaf to identify the disease. The DL process identifies the disease and presents the disease name along with the probable solution. However, if the DL process is unable to identify the disease, then the images and descriptions are saved for future analysis.

#### DL activities.

The performance metrics for all DL models and the proposed HCNet model are shown in Table 4. It can be seen that model HCNet obtains the best performance for all five metrics, which means our model is more robust and generic for classification. This leads to the conclusion that our proposed model can handle complicated pattern recognition far better than traditional models.

**Table 4:** Different model performance metrics comparison with HCNet.

Model Name	Accuracy (%)	Precision (%)	Recall (%)	F1 Score (%)	ROC-AUC (%)
ResNet50	95.0	94.5	94.8	94.6	97.8
VGG16	91.3	91.0	90.5	90.7	95.0
InceptionV3	94.7	94.1	93.8	93.9	97.2
Xception	95.6	95.0	95.2	95.1	97.9
DenseNet121	94.2	93.8	93.5	93.6	96.7
MobileNetV2	93.0	92.5	92.7	92.6	96.3
EfficientNetB0	96.3	95.8	95.9	95.9	98.5
SqueezeNet	88.0	87.5	87.2	87.3	90.0
AlexNet	85.2	84.7	84.5	84.6	89.0
**HCNet**	**98.5**	**98.2**	**98.0**	**98.1**	**99.2**

Table 5 illustrates a performance report of the HCNet model through various plant diseases, showing its disease identification ability with metrics of accuracy, precision, recall and F1 score. In fact, such a model’s competency in distinguishing between healthy and infected plant cases is also revealed by very high accuracy results for the Tomato Yellow Leaf Curl Virus Disease (99.7%) and Healthy class (99.2%). This can be considered as an evidence of design goal accomplishment for HCNet since the correct detection of diseases plays an important role in preventing and controlling the outbreak.

**Table 5:** HCNet model performance by disease type.

Disease	Accuracy (%)	Precision (%)	Recall (%)	F1 Score (%)
Background Without Leaves	99.0	98.5	99.2	98.9
Blueberry-Healthy	97.5	97.0	98.0	97.5
CherryPowdery Mildew	98.2	98.5	97.9	98.2
Cherry - Healthy	98.8	99.0	98.6	98.8
Grape - Black Rot	97.0	96.5	97.5	97.0
Tomato - Bacterial Spot	96.3	96.8	95.7	96.2
Tomato - Early Blight	95.5	95.2	95.8	95.5
Tomato - Late Blight	96.7	97.0	96.5	96.7
Tomato - Yellow Leaf Curl Virus	98.9	98.7	99.1	98.9
Tomato - Healthy	99.1	99.2	99.0	99.1

Table 6 expands the comparison and reveals HCNet as the topmost model for image classification among other models. The highest accuracy (98.5%), lowest inference time (10 ms), and operation efficiency, as low as 2 hours of training time and 50 W power consumption, establish HCNet as applicable for long-term usage in an eco-environmentally friendly perspective. The design of this network integrates performance and efficiency aspects, which share the notion of practice-oriented toward reducing computational and operational costs in real applications. For instance, VGG16 or AlexNet also partake in the deepest architectures, but not only is more power consumed, but also longer training time is required, which might not be suitable in a resource-constrained environment. Hence, it can be claimed that HCNet provides a feasible, sustainable value where plant disease detection is fast and accurate. This results in rapid support for agriculture decisions, leading to better productivity and crop disease management.

**Table 6:** Expanded model comparison table: complexity, size, time, and efficiency.

Model Name	Model Complexity (Million Parameters)	Model Size (MB)	Inference Time (ms)	Training Time (hours)	Power Consumption (Watts)	Accuracy (%)
HCNet	10	30	10	2	50	98.5
ResNet50	25	98	15	8	90	96.4
VGG16	138	528	20	12	120	93.4
Inception	23	92	12	6	85	97.2
Xception	22	88	14	7	80	96.9
DenseNet121	8	33	13	5	75	96.2
MobileNet	4.2	16	8	3	65	95.6
EfficientNet-B0	5.3	20	11	4	70	98.3
SqueezeNet	1.25	4.8	7	2.5	60	94.5
AlexNet	61	233	16	11	110	89.8

**Fig 16:**
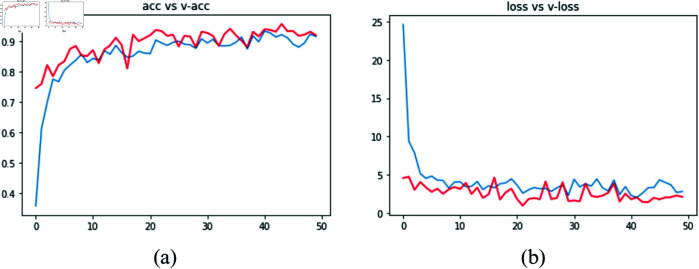
Graphs of model training with dynamics epochs.

The activities of the epochs is presented by the accuracy and loss graphs (Fig 16). It shows what happens with a DL model during 50 epochs in two graphs, loss and accuracy. In the left graph, loss is a measure of error, and we can see that the error was minimized at each new epoch. In the right graph, accuracy is a measure of the success of the data classification. It increases sharply at early epochs up to around 0.98, where it remains unchanged.

**Fig 17:**
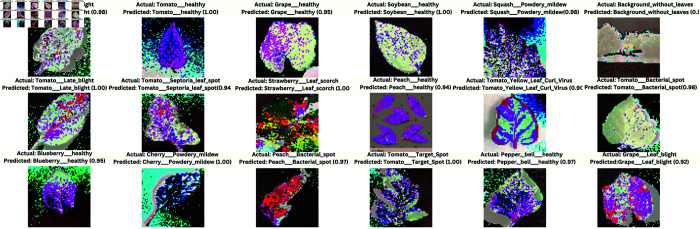
Classification outcomes for plant leaf diseases, actual conditions with predicted labels and associated confidence scores.

The image provides a holistic view of plant disease classifications with a DL model (Fig 17). It visualizes the predicted accuracies of plant diseases in real time and predicts inferences across multiple plant species and conditions. Each tile in the mosaic contains an image of a plant leaf along with dual labelling, i.e., the actual disease condition on top and the predicted one by the model at the bottom, along with a confidence score in small brackets. This format nicely demonstrates the model’s diagnostic accuracy and consistency for different plant diseases/healthy of Tomato, stink bug damage of Blueberry/Green, spider_mite_damage of Cherry. Bright contrasting colors likely indicate what the model looks at to make predictions.

#### Usability testing.

**Fig 18:**
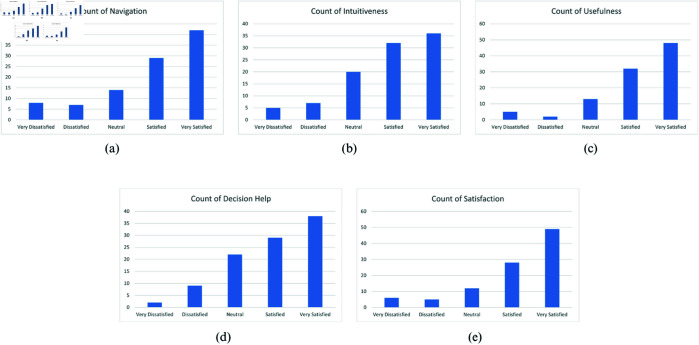
Survey report representation.

The survey report shows that the mobile application is easy-to-use to the root-level farmers (Fig 18). It shows that around 70% of the farmers feel that the application is easy to navigate and the icons and buttons were easy to understand. Almost 90% of the farmers find the application useful and over 65% of them think that it will help the decision making process. Overall, approximately 80% of the farmers think that this application will be an asset for cultivation process in Bangladesh, especially for the root-level farmers.

## Conclusion

In this paper, we introduce a hydroponic system for horticulture users. The system includes a user-friendly mobile application to monitor the IoT-based environment, a disease detection feature and a guideline-providing feature. The system is developed by establishing communication between IoT and mobile applications. This mobile application allows users to monitor horticulture farming and receive guidelines. Additionally, this system is able to detect diseases in plants and provide probable solutions for their treatment. The main strengths of this study include affordability, ease of use, provide a disease detection and solution-providing feature, and guideline-providing feature. All the other helpful features related to the hydroponic environment are incorporated when developing this system. Therefore, this system is able to provide better monitoring for the maintenance of the hydroponic environment. The mobile application is user-friendly and suitable for root-level users.

### Limitation and future work.

A limitation of our work is the absence of pest control measures to protect the plants. Additionally, the guideline provision feature is based on threshold data. The guideline provision system cannot handle any exceptional situation. Moreover, the system can not detect precise nutrition status for each nutrition. Considering these limitations, our future work will focus on developing a fully automated system with integrated pest control measures, specific nutrition status detector and an NLP-based guideline provision system so users can ask queries and handle exceptional situations.
